# True Anaphylaxis Due to an Amide-Type Local Agent: A Case Report

**DOI:** 10.7759/cureus.89655

**Published:** 2025-08-08

**Authors:** Hikaru Matsumoto, Akira Nemoto, Yukitoshi Niiyama

**Affiliations:** 1 Department of Anesthesiology and Intensive Care Medicine, Akita University Graduate School of Medicine, Akita, JPN

**Keywords:** allergy and anaphylaxis, case report, cross-activity, local anesthesia, pure lidocaine

## Abstract

Local anesthetics (LAs) are widely used to relieve surgical pain. Pure amide-type LAs rarely cause allergic reactions. Here, we present a case of anaphylaxis induced by multiple pure amide agents. We report a case of a 17-year-old girl with no history of allergies who was scheduled to undergo orthopedic surgery. She presented with anaphylactic symptoms after receiving peripheral nerve blocks. Postoperative allergy tests were positive for two kinds of amide-type LAs: pure lidocaine and ropivacaine. In conclusion, cross-reactivity should be considered in case of an allergic reaction to an amide-type agent.

## Introduction

Local anesthetics (LAs) are used in various fields of medicine to reduce surgical pain. They are divided into two main groups: amides and amino esters. In France, 210017 adverse reactions caused by LAs were reported for 12 years. Although many reports of LA-induced adverse events have been published, reports of true LA-induced allergies are rare. Allergies have been recognized to be mainly associated with amino-ester LAs because of a derivative of para-aminobenzoic acid; conversely, allergies have been rarely related to the use of amide LAs. A large clinical investigation estimated the prevalence of LA-induced allergies to be approximately 1% [1]. However, there is a possibility of overestimation, because recent literature suggests that the prevalence is lower [2]. Although there are lots of lidocaine-induced allergy reports due to its use in various medical fields, most of the allergic reactions are caused by the additive drug. Thus, pure lidocaine that contains no pH adjusters or preservatives (methylparaben) rarely causes an allergic reaction. Here, we describe a case of a patient who developed anaphylaxis possibly caused by pure lidocaine; ropivacaine might also have been causative as a cross-reactive agent.

## Case presentation

**Figure 1:**
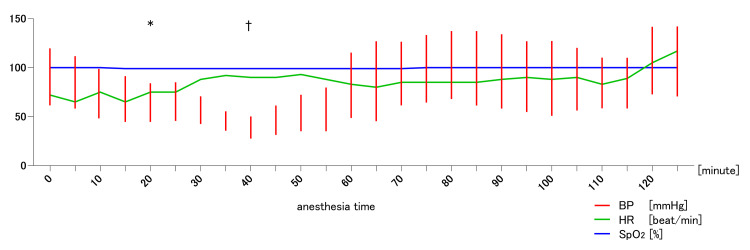
Vital sign trend in the first surgery. *: peripheral nerve blocks by a mixture of 75 mg ropivacaine and 100 mg lidocaine †: urticarial and adrenaline intramuscular administration

The patient was a 17-year-old girl with no remarkable LA-related medical, allergic, or exposure history who injured her left knee while playing basketball. She visited the Department of Orthopedics because of knee joint instability and swelling. Magnetic resonance imaging revealed a left anterior cruciate ligament (ACL) injury. The patient decided to undergo ACL reconstruction. No abnormalities were observed during a preoperative examination. Surgery was performed four months after the injury by using a combination of general anesthesia and peripheral nerve blocks (Figure 1).

**Figure 2:**
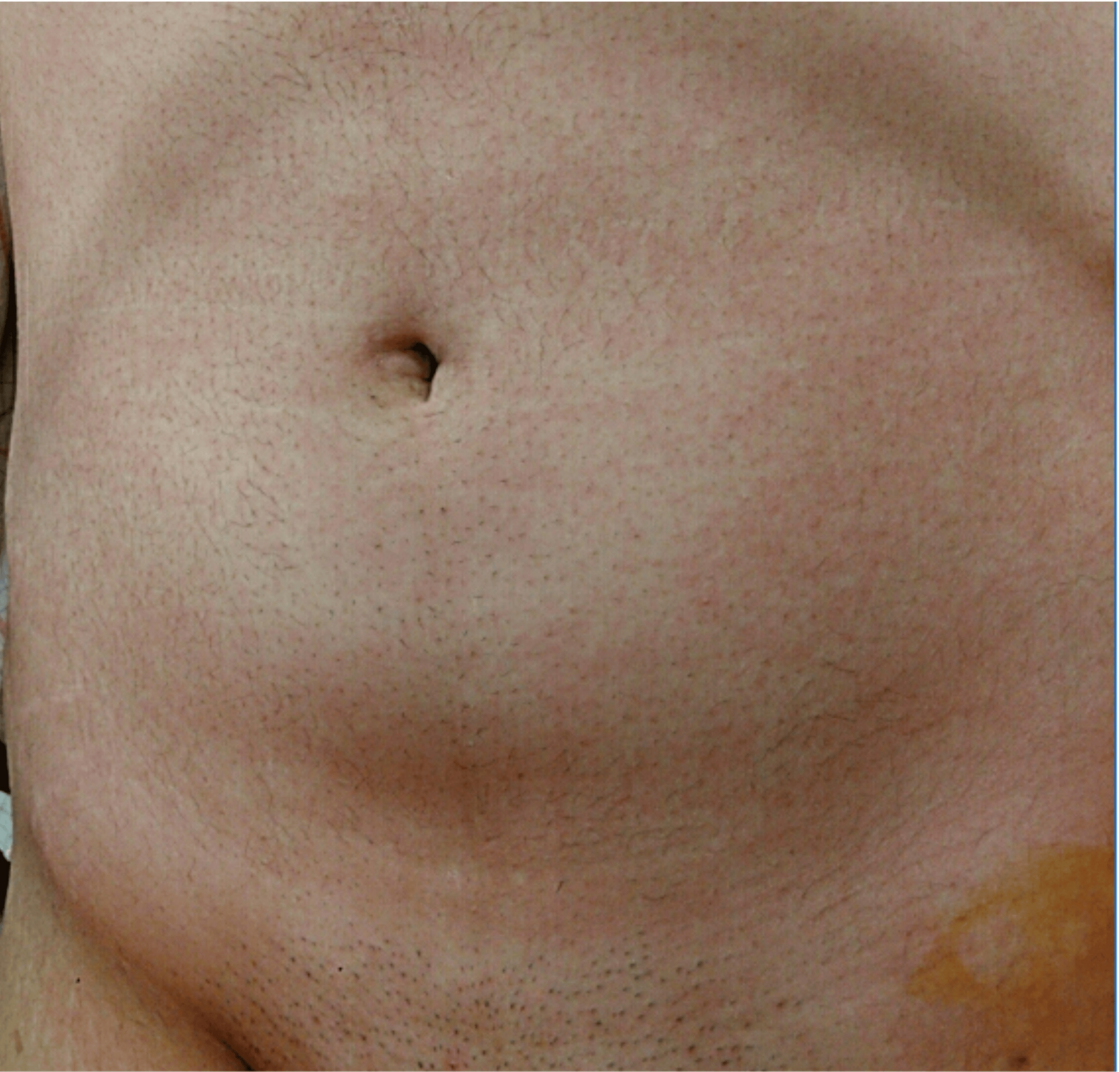
Abdominal urticaria in the first surgery. Abdominal urticaria spread throughout the body immediately after the peripheral nerve block.

Anesthesia was induced with intravenous (IV) infusion of propofol (target-controlled infusion 4 μg/ml), remifentanil (0.4 μg/kg/min) IV, and rocuronium bromide (60 mg) IV for neuromuscular blockade. Anesthesia was maintained by a continuous IV propofol infusion (3.5 μg/ml) and remifentanil (0.1 μg/kg/min) to achieve a bispectral index (BIS) of 40 to 60 on the BIS monitor (Aspect Medical Systems, Newton, MA). After induction, peripheral nerve blocks and surgical antimicrobial prophylaxis with IV piperacillin 1000 mg were administered. Regarding peripheral nerve blocks, a mixture of 75 mg ropivacaine (Sandoz Pharma Inc., Tokyo, Japan) and 100 mg lidocaine (Sandoz Pharma Inc.) was used for femoral and sciatic nerve blocks. Ten minutes after the nerve block, the patient developed hypotension, and tachycardia occurred, which gradually worsened to 55/30 mmHg and 95 beats/min, respectively. Twenty minutes after the nerve block, the patient developed urticaria on the abdomen, which spread throughout the body (Figure 2).

Although there was no airway obstruction, a diagnosis of anaphylaxis was made. A single 0.3 mg adrenaline (Daiichi Sankyo Co., Ltd., Tokyo, Japan) intramuscular injection immediately improved circulation and skin symptoms; additional adrenaline administration was not needed. The surgery was postponed and the patient was moved to the intensive care unit (ICU) after recovering from general anesthesia. She did not develop biphasic anaphylaxis in the ICU. Blood samples obtained 2 h after the reaction indicated normal levels of histamine and increased β tryptase. Blood samples could not be obtained in the earlier and later phases.

Although a skin prick test was proposed to determine which LA was the allergen, the patient’s parents did not accept our proposal and insisted on an in vitro test to avoid exposure to the allergic agent. Two months after the surgery, the patient underwent a drug-induced lymphocyte stimulation test (DLST) and specific immunoglobulin E (sIgE) examination (view39, Thermo Fisher Scientific Inc., Tokyo, Japan) as an in vitro test (Table 1).

**Table 1:** In vitro and vivo allergic examination after surgery. DLST: Drug-induced lymphocyte stimulation test; sIdE test: specific immunoglobulin-E antibody test. We considered that more than 50% of the histamine (positive control) diameter was positive. Pure lidocaine was used in the DLST and the prick test.

	Ropivacaine	Lidocaine	Rocuronium	Propofol	Piperacilline	Latex
DLST	+	-	-	-	-	
sIgE test						-
Skin Prick Test	-	+	-		-	-

The DLST was positive only for ropivacaine. A sIgE examination showed a negative reaction to latex antigen. Therefore, general anesthesia and peripheral nerve blocks without ropivacaine were administered for ACL reconstruction. To avoid the risk of methylparaben allergy and extend the pharmacological effect of nerve blocks, pure lidocaine (Terumo Co., Ltd., Tokyo, Japan), which did not contain methylparaben or any additive agent but adrenaline, was used for nerve blocks; that is, 150 mg lidocaine with 0.2 mg adrenaline (20 ml of 100,000× diluted adrenaline solution) was administered. Twenty minutes after the nerve blocks, the patient developed urticaria on the abdomen without hypotension or airway obstruction. The urticaria immediately disappeared after the IV administration of 5 mg chlorpheniramine (Takata Pharmaceutical Co., Ltd., Saitama, Japan). Since the patient did not show anaphylactic symptoms, surgery was performed entirely after the rash disappeared. To prevent the recurrence of anaphylaxis, a further allergy test, which was a skin prick test, was administered after obtaining the patient’s and her parents’ consent. The test was performed six weeks after the second surgery. The patient developed wheals in response to histamine as the positive control, and pure lidocaine (diameters, 8 and 4 mm, respectively). Saline was used as the negative control. The patient did not develop wheals on exposure to other perioperative drugs (diameters, 2 mm and 1 mm in the ropivacaine and the other drugs, respectively) nor did she show any anaphylactic signs during the skin test (Table 1). It was essential to conduct the skin test to get a final diagnosis about an anaphylaxis agent.

## Discussion

Herein, we report a case of a 17-year-old girl who developed anaphylaxis on exposure to an amide-type LA. We had two considerations. First, lidocaine allergy was mostly caused by methylparaben, which is used as a preservative agent. Thus, this was a case of pure lidocaine allergy, which is extremely rare. Second, the DLST was positive for ropivacaine, and the skin prick test was positive for lidocaine. Amide-type LAs can cause multiple allergies.

Methylparaben is added as a preservative to confer lidocaine with antibacterial effects. Lidocaine used in the first surgery contained 0.1% methylparaben, and ropivacaine did not contain it. Although the DLST was not positive for lidocaine, it has low sensitivity for detecting anaphylaxis causative agents [3]. Therefore, we should have recognized that a negative DLST cannot completely exclude suspected drugs. In the second surgery, we planned to use pure lidocaine as a peripheral nerve blocking agent in case methylparaben acted as an allergen. However, lidocaine has a short blocking effect; therefore, we added 100,000× diluted adrenaline to prolong the pharmacological nerve blocking effect. Adrenaline in nerve blocks may act as an antiallergic agent. Many reports on LA allergies and adverse events have been published in various fields of medical care. However, in those 2978 reports, the prevalence of confirmed true IgE-mediated pure LA allergy has been reported to be less than 1% [2]. IgE-mediated allergy was established as the final diagnosis based on a skin prick or intradermal test [2]. Hence, this case presentation of pure lidocaine-induced anaphylaxis is extremely rare.

In vitro examinations, i.e., the sIgE test and DLST, could not identify lidocaine as the causative anaphylactic drug. Although the former test can result in the identification of a suspected drug, a negative reaction cannot be used as confirmation to exclude a suspected drug owing to its low specificity. Because the latter is mainly used to diagnose type IV allergic drugs, detecting type I reactions was unsuitable. However, the DLST can sometimes show a positive reaction to type I allergic drugs because some patients have both type I and IV allergies to the same agent [4] or provide a false-positive result [3]. Additionally, cross-reactivity occurs between amide agents [2]. The prick test is performed by diluting the suspect agent and then non-diluted [5]. Further investigation of cross-reactivity requires an intradermal test or a repeat skin prick test using a non-diluted suspect drug to obtain a final diagnosis when the skin prick test is negative. According to these two anesthesia management methods, immediate reactions would occur in IgE-mediated allergy caused by pure lidocaine, and further examinations are needed to reveal whether ropivacaine acts as a type IV and/or I allergic or false-positive agent. To determine the effect of ropivacaine, the patient will have to undergo an additional patch test and an intradermal test to confirm a type IV and I final diagnosis, respectively.

## Conclusions

In conclusion, exposure to pure lidocaine induced a perioperative anaphylactic reaction in our patient. Although pure lidocaine rarely causes an allergic reaction, in case of lidocaine usage, we should bear in mind that it is a suspected allergic agent. In addition, in cases where LA allergy is suspected, cross-reactivity of amide-type LAs must be considered.

## References

[1] Fuzier R, Lapeyre-Mestre M, Mertes PM (2009). Immediate- and delayed-type allergic reactions to amide local anesthetics: clinical features and skin testing. Pharmacoepidemiol Drug Saf.

[2] Bhole MV, Manson AL, Seneviratne SL, Misbah SA (2012). IgE-mediated allergy to local anaesthetics: separating fact from perception: a UK perspective. Br J Anaesth.

[3] Saito M, Yagi M, Uno K, Takanaka K (2008). Comparative study of the usefulness of the drug-induced lymphocyte stimulation test and the leukocyte migration test in drug allergies. Biol Pharm Bull.

[4] Cederbrant K, Marcusson-Stâhl M, Hultman P (2000). Characterization of primary recall in vitro lymphocyte responses to bacampicillin in allergic subjects. Clin Exp Allergy.

[5] Thyssen JP, Menné T, Elberling J, Plaschke P, Johansen JD (2008). Hypersensitivity to local anaesthetics--update and proposal of evaluation algorithm. Contact Dermatitis.

